# Internal mammary regional management after neoadjuvant therapy in breast cancer

**DOI:** 10.1097/JS9.0000000000001188

**Published:** 2024-02-16

**Authors:** Zhao Bi, Chun-Hui Zheng, Tong-Yue Ren, Yong-Sheng Wang

**Affiliations:** Shandong Cancer Hospital and Institute, Shandong First Medical University and Shandong Academy of Medical Sciences, Jinan, Shandong, People’s Republic of China

## Introduction

Highlights

- The definition of nodal pathological complete response after neoadjuvant therapy (NAT) needs to include the status of the axillary lymph node and the internal mammary lymph node.
- Internal mammary regional management might be inadequate without assessment of internal mammary lymph nodes.
- Survival benefit generated by internal mammary nodal irradiation would be greatly diluted by the population with negative internal mammary lymph nodes after NAT.
- Internal mammary-sentinel lymph node biopsy after NAT could help to identify the metastasis of internal mammary lymph nodes after NAT in order to guide internal mammary nodal irradiation after NAT.

Neoadjuvant therapy (NAT) is currently administered to patients with locally advanced breast cancers or to early-stage breast cancer having an indication of systemic therapy^1^. A major clinical benefit of NAT is the downstaging of the tumor. As a result, patients with clinical nodal positive (cN+) disease might have more chance to achieve nodal pathological complete response (pCR) and omit axillary lymph node dissection (ALND) after NAT. At the same time, NAT could also help to guide individualized regional nodal irradiation (RNI). The axillary treatment after NAT has been consensus, but the internal mammary regional management after NAT is still controversial. The nodal pCR after NAT is defined as no metastatic carcinoma remaining in the nodes^2^. This definition of nodal pCR only focuses on the pathological state of the axillary lymph node (ALN) while overlooks the internal mammary lymph node (IMLN). Internal mammary nodal irradiation (IMNI) is one of the most important treatments in IMLN. However, the current indication for IMNI (i.e. high risk of IMLN metastasis without histopathological confirmation of IMLN metastasis) has the potential to result in overtreatment or undertreatment^3^. Therefore, the value of IMLN metastasis information in guiding IMNI is much greater than the high-risk factors of IMLN metastases. Here, we will discuss the optimal management of the internal mammary region after NAT.

## The nodal pCR after NAT needs include the pathological status of ALN and IMLN

The pCR after NAT has been shown to be associated with improved survival outcomes, and nodal pCR is defined as no tumor cells or metastatic carcinoma remaining in the nodes^2^. This definition only focuses on the pathological status of ALN while overlooks the IMLN, which is one of the most important metastatic pathways of breast cancer. IMLN metastasis has similar prognostic importance as that of ALN, and the lymphatic metastasis and staging should involve not only ALN but also IMLN. For patients without NAT, the incidence of IMLN metastasis was 18–33%, with 2–11% of patients whose lymph node metastases were situated only in IMLN without ALN. For patients receiving NAT, the incidence of IMLN metastasis was 7.1–7.3%^3^. There were still some patients whose node metastases were situated only in IMLN without ALN after NAT. The pathology stage after NAT might also be changed when combining the status of ALN and IMLN. Therefore, the pathological status of IMLN should be taken into account in order to achieve perfect nodal pCR definition. In clinical practice, the scope of nodal surgery after NAT also includes the internal mammary region.

## Internal mammary regional management might be undertreatment without IMLN assessment

IMNI is one of the main management of the internal mammary region. With the continuous maturation of radiotherapy technology, IMNI has improved the survival of breast cancer patients and gained an increasing amount of attention (Table 1). However, the benefits of IMNI may also be diluted to some extent by increasing local control when systemic therapy is effective. So, it is very important to grasp the indication of IMNI accurately^4^. At present, the indication of IMNI without NAT mainly depends on the high-risk factors of IMLN metastases, such as positive axillary nodes. According to extended radical mastectomy data, IMLN metastasis occurred in 9.2% of patients with no positive axillary nodes, 19.6% of patients with one to three positive axillary nodes, and 38.3% of patients with more than three positive axillary nodes^4^. The NCCN guideline also recommends IMNI for patients with more than three positive ALNs (category 1) and strongly suggests IMNI for patients with one to three positive ALNs (category 2A)^5^.

**Table 1:** Survival benefit of IMNI.

Items	French trial	EORTC 22922/10925	MA.20	DBCG-IMN
Patients (*n*)	1334	4004	1832	3089
Follow-up (median)	11.3 years	15.7 years	9.5 years	14.8 years
Proportion of ALN+	75.2%	55.2%	90.3%	100%
Breast cancer recurrence	43.1% vs. 50.3%	24.5% vs. 27.1%	NA	NA
Breast cancer mortality	NA	16.0% vs. 19.8%	10.3% vs. 12.3%	31.7% vs. 33.9%
DFS	53.2% vs. 49.9%	60.8% vs. 59.9%	82.0% vs. 77.0%	NA
OS	62.6% vs. 59.3%	73.1% vs. 70.9%	82.8% vs. 81.8%	60.1% vs. 55.4%

ALN, axillary lymph node; DFS, disease-free survival; IMNI, internal mammary nodal irradiation; NA, not applicable; OS, overall survival.

In patients with NAT, the indication and target volumes for radiotherapy need to be individualized based on the initial tumor stage and the tumor's response to treatment^6^. The NCCN (National Comprehensive Cancer Network) guideline recommends that in patients treated with NAT, adjuvant radiotherapy is based on the maximal disease stage (i.e. clinical stage, pathologic stage, and tumor characteristics) at diagnosis and pathology results after NAT^5^. The 2023 St. Gallen International Consensus Conference strongly endorsed that the extent of RNI could be individualized by several risk factors: (1) lowest-risk patients (cN0 at baseline with nodal pCR after NAT) requiring no RNI; (2) intermediate-risk patients (cN1 at baseline with nodal pCR after NAT; or without ALND) receiving exclusive levels 1–2 axillary radiotherapy; (3) highest-risk patients (cN2–3 at baseline with nodal pCR after NAT; or those with residual nodal involvement after NAT) receiving RNI to levels 1–3 ALN, and to supraclavicular and IMLN^7^. However, the recommendation of the 2023 St. Gallen International Consensus Conference might not be perfect. This recommendation only focuses on the pathological state of ALN while overlooking the IMLN. For patients with IMLN+/ALN− with intermediate/lowest risk after NAT, the treatment might be inadequate without IMNI. The NSABP B-51 trial evaluated whether RNI significantly improves survival in cN1 patients who are found to be ypN0 after NAT^8^. The first result of the B-51 trial showed that RNI after surgery did not improve the 5-year invasive breast cancer recurrence-free interval, disease-free survival (DFS), or overall survival (OS). These findings suggested that downstaging involved axillary nodes after NAT can optimize adjuvant radiotherapy use without adversely affecting oncologic outcomes. However, the inclusion criteria of the B-51 trial only focused on the status of ALN after NAT. There might still be some patients with IMLN+/ALN− after NAT. In other words, the regional control might be inadequate for these patients. Therefore, the design of RNI needs to be combined with the status of ALN and IMLN after NAT.

## Benefit of IMNI might be diluted by negative IMLN after NAT

A multicenter, retrospective study (KROG 16-14) enrolled 217 patients with IMLN metastases, which were defined as involved in imaging methods (measuring >0.5 cm)^9^. All patients received NAT, followed by breast surgery. With a median follow-up duration of 61 months, the recurrence in IMLN was 2.8% and 13.0%, respectively, with or without IMNI. Kim *et al*.^10^ also retrospectively assessed the survival outcome of IMNI in breast cancer patients with clinically overt metastasis to the IMLN. The metastasis to the IMLN was defined as IMLN measuring greater than 0.5 cm on breast MRI before the initiation of NAT. The result showed that even without an IMLN dissection after NAT, most patients were IMLN failure-free with an IMNI.

However, the survival benefit generated by IMNI would be greatly diluted by the population with negative IMLN after NAT. One retrospective study assessed the survival benefits of 537 patients with IMLN biopsy^11^. The results showed that adding IMNI did not improve DFS (*P*=0.099) or OS (*P*=0.486) in patients with negative IMLN, but it did significantly increase the risk of radiation-induced lung injury. For patients with positive IMLN disease, adding IMNI improved 5-year DFS (87.3% vs. 52.5%, *P*=0.040) but had no effect on OS (*P*=0.603). Therefore, the actual metastasis information of IMLN after NAT had a better value in guiding IMNI after NAT, considering the high risk of radiation-induced lung injury. Unfortunately, IMLN usually has a deep anatomical position and small diameter (<0.5 cm); the sensitivity of iconography methods cannot meet the clinical requirements to detect IMLN metastases. So, one minimally invasive diagnostic technique was needed to assess accurately the status of IMLN metastasis.

## Internal mammary-sentinel lymph node biopsy (IM-SLNB) could assess accurately the status of IMLN after NAT

With the development of the sentinel lymph node biopsy (SLNB) technique, IM-SLNB is expected to be a promising technique in assessing the status of IMLN^12^. However, there is still controversy about whether IM-SLNB should be carried out regularly. On the one hand, the traditional radiotracer injection technique had a very low detection rate (average 13%, 0–37%) of internal mammary sentinel lymph node (IMSLN); on the other hand, most clinical trials of IM-SLNB still use indications of axillary SLNB (only implemented in cN0 patients). The low metastasis rate of IMLN results in little influence on nodal staging and local/system treatment decisions, significantly reducing the clinical application value of IM-SLNB^4^. At the same time, the implementation of IM-SLNB in routine clinical practice is also complicated by anatomical issues (high variability of IMLN and internal mammary lymphatic drainage) and technical complications in the surgical procedure.

Qiu *et al*.^12^ injected radiotracer using a modified injection technique (peri-areolar intra-parenchymal, high volume, and ultrasound guidance), which could significantly improve the preoperative visualization rate of the IMSLN. This new technique laid a technical feasibility for the further study and clinical application of IM-SLNB. According to the previous studies, the visualization rate of the IMSLN after NAT was 31.8–56.8%. The incidence of complications of IM-SLNB was 7.5–9.4%, including internal mammary artery injury and pleural injury. And these complications were within the acceptable range. Therefore, the IM-SLNB after NAT could also be carried out regularly in patients with IMLN lymphoscintigraphy in order to guide staging and more accurate IMNI strategy. For patients who cannot receive IM-SLNB, the nomogram developed by Qiu *et al*.^12^ could estimate the individual likelihood of having IMLN metastasis. This can also assist radiation oncologists in making individualized selections of patients who would benefit from IMNI.

In conclusion, the pathological status of IMLN should also be taken into account in order to achieve a perfect nodal pCR definition. The actual metastasis information of IMLN after NAT had a better value in guiding IMNI after NAT. IM-SLNB after NAT could also be carried out regularly in patients with IMLN lymphoscintigraphy in order to guide staging and more accurate IMNI strategy.

## Ethical approval

Not applicable.

## Sources of funding

Shanghai Anti-cancer and Anti-cancer Development Foundation (CYBER-2022-002) and China Postdoctoral Science Foundation (Grant No. 2022M721987).

## Author contribution

Z.B. and Y.-S.W.: wrote the paper. All authors read and approved the final manuscript.

## Conflicts of interest disclosure

All authors have stated that they have no conflicts of interest in this work.

## Research registration unique identifying number (UIN)

1. Name of the registry: not applicable.
2. Unique identifying number or registration ID: not applicable.
3. Hyperlink to your specific registration (must be publicly accessible and will be checked): not applicable.

## Guarantor

The guarantors are Zhao Bi and Yong-Sheng Wang.

## Data availability statement

Not applicable.
